# ‘Venus trapped, Mars transits': Cu and Fe redox chemistry, cellular topography and *in situ* ligand binding in terrestrial isopod hepatopancreas

**DOI:** 10.1098/rsob.150270

**Published:** 2016-03-02

**Authors:** P. Kille, A. J. Morgan, K. Powell, J. F. W. Mosselmans, D. Hart, P. Gunning, A. Hayes, D. Scarborough, I. McDonald, J. M. Charnock

**Affiliations:** 1 Cardiff School of Biosciences, Cardiff University, Park Place, Cardiff CF10 3US, UK; 2 Diamond Light Source Ltd, Harwell Science and Innovation Campus, Didcot, UK; 3 Smith and Nephew, Heslington, York Science Park, York YO10 5DF, UK; 4 School of Earth and Ocean Sciences, Cardiff University, Park Place, Cardiff CF10 3AT, UK; 5 School of Earth, Atmospheric and Environmental Sciences, University of Manchester, Williamson Building, Oxford Road, Manchester M13 9PL, UK

**Keywords:** iron, copper, speciation, µ-focus, synchrotron, isopod

## Abstract

Woodlice efficiently sequester copper (Cu) in ‘cuprosomes' within hepatopancreatic ‘S' cells. Binuclear ‘B’ cells in the hepatopancreas form iron (Fe) deposits; these cells apparently undergo an apocrine secretory diurnal cycle linked to nocturnal feeding. Synchrotron-based µ-focus X-ray spectroscopy undertaken on thin sections was used to characterize the ligands binding Cu and Fe in S and B cells of *Oniscus asellus* (Isopoda). Main findings were: (i) morphometry confirmed a diurnal B-cell apocrine cycle; (ii) X-ray fluorescence (XRF) mapping indicated that Cu was co-distributed with sulfur (mainly in S cells), and Fe was co-distributed with phosphate (mainly in B cells); (iii) XRF mapping revealed an intimate morphological relationship between the basal regions of adjacent S and B cells; (iv) molecular modelling and Fourier transform analyses indicated that Cu in the reduced Cu^+^ state is mainly coordinated to thiol-rich ligands (Cu–S bond length 2.3 Å) in both cell types, while Fe in the oxidized Fe^3+^ state is predominantly oxygen coordinated (estimated Fe–O bond length of approx. 2 Å), with an outer shell of Fe scatterers at approximately 3.05 Å; and (v) no significant differences occur in Cu or Fe speciation at key nodes in the apocrine cycle. Findings imply that S and B cells form integrated unit-pairs; a functional role for secretions from these cellular units in the digestion of recalcitrant dietary components is hypothesized.

## 1. Introduction

Approximately 30% of all proteins are considered to require a metal cofactor, usually a transition metal such as Cu, Fe, Mn or Zn [1]. Moreover, metal ions and proteins are also functionally interdependent in other ways, including metal-mediated control of gene expression [2], direct [3] and indirect [4] metal ion involvement in intracellular signalling, and the roles of certain proteins as metallochaperones [5] and metallotransporters [6]. The requisite selectivity of these molecular events crucially depends on discriminatory metal sensors [7]. It is almost inevitable that imbalances in the homeostasis of these essential transition metals can lead to cytotoxicity and disease processes in both invertebrates and vertebrates because surpluses of redox-active species often induce reactive oxyradical generation [8]. For example, elevated levels of Fe are associated with neurodegenerative conditions such as Alzheimer's disease [9], while the progressive liver degeneration in the genetic disorder Wilson's disease is characterized by gross Cu deposition within hepatocytes [10]. Thus, cells and organisms are both beneficiaries and hostages of the coordination chemistries and redox properties of metabolically essential transition metals.

In general, essential and non-essential metals are heterogeneously distributed among biological tissues and are often compartmentalized within individual cells as a function of their identities, life-stage or health [11,12]. Significant insights into the physiology and pathophysiology of metals can potentially, therefore, be provided by bio-imaging techniques [13–15]. While technically challenging, combining the mapping of metals within subcellular compartments and simultaneously determining their chemical states (i.e. ‘speciation’) is a necessary prelude to better understanding metal homeostasis [2]. Synchrotron-based X-ray absorption spectroscopy (XAS) bio-imaging, in different modes, can map metal distributions in a spatially resolved manner, as well as providing information about oxidation states and covalence numbers. In addition, this family of intense brightness techniques can furnish structural information about the identities of neighbouring atoms, as well as information about the bond lengths separating neighbouring atoms from the metal atom cores being probed spectroscopically [16], unique properties beyond the capabilities of X-ray analyses in electron and proton probe instruments [13].

Molecular-genetic evidence indicates that Cu and Fe transport and homeostatic pathways are highly conserved from yeast to mammals [17,18]*.* A corollary of this statement is that the interdependent features of the Cu and Fe transport networks in mammals are illuminated by observations in lower organisms. This principle has motivated major research efforts on Cu and Fe metabolism in yeast as a model system [8,19], but has not hitherto engendered equivalent detailed studies on the diverse, often highly discriminating, intracellular metal-sequestering organelles of invertebrates. We propose to address this shortcoming by describing spatially resolved Cu and Fe redox states as well as ligand-binding speciation in the midgut (hepatopancreas) of terrestrial isopods, whose constituent cells offer an impressive example of transition metal specificity, partitioning and homeostasis.

Terrestrial isopods (suborder Oniscidae; commonly and variously referred to as ‘woodlice’, ‘sowbugs' and ‘slaters') are the most successful crustacean land colonizers [20]. They have long been recognized as generalist detritivores [21], but recent observations suggest that they warrant the status of keystone fungal grazers in temperate woodland habitats [22]. The taxon evolved in shallow seas during the Early to Mid-Palaeozoic era (*ca* 541–440 Ma), a period during which oxidation conditions had resulted in Fe availability in seawater plummeting with concomitant rise in Cu availability [23]. It is highly plausible that the respiratory pigment of isopods, haemocyanin, evolved from phenoloxidase, a type 3 Cu protein, with the conversion from enzymatic to oxygen-binding functions being facilitated through occlusion of the catalytic site by a peptide domain [24]. Oxygen binding by haemocyanin involves pairs of Cu atoms becoming oxidized from Cu (I) to Cu (II). Haemocyanin is synthesized in a four-lobed tubular hepatopancreas, an organ containing the highest soft tissue Cu concentration recorded in any terrestrial animal under physiological conditions [25], a storage level that is orders of magnitude higher than that required to satisfy direct respiratory demands [26]. Cu assimilation efficiency and storage capacity in isopods evidently increases with the degree of adaptation to terrestrial habitats, perhaps because Cu is at least periodically difficult for woodlice to acquire [27].

Numerous studies have shown that the woodlouse hepatopancreas comprises two distinct cell types in roughly equal numbers and possibly forming functionally integrated units: (i) small conical ‘S' cells that are mainly absorptive, and basally contain numerous discrete Cu-storing organelles, the cuprosomes, with an S-donating matrix; and (ii) large binucleate ‘B’ cells projecting into the organ's lumen that are involved in absorption and secretion, and contain glycogen, prominent lipid droplets and multivesicular organelles with floccular Fe deposits sequestered [28–31] within a phosphate-rich matrix [25,28–31]. The half-life of S cells and their cuprosomes is relatively long, with some authors (e.g. [32]), but not all [33], claiming that Cu loss is negligible even in woodlice consuming a Cu-impoverished diet. The nature of the Cu-binding ligand chemistry of cuprosomes has not hitherto been described; Donker *et al.* [34] concluded that *Porcellio scaber* hepatopancreas may not contain thiol-rich metallothionein (MT), although Žnidaršič [35] did identify MT-like protein in the hindgut of the same woodlouse species. It is reasonable to hypothesize that cuprosomes not only immobilize Cu to protect the storage cell from the potential toxicity of this redox-active metal, but must also be able to release Cu to serve haemocyanin synthesis and phenoloxidase-related nutritional and immune function requirements. The cytology and metallome of B cells are even less well understood than those of S cells. Hames & Hopkin [36] reported that B cells of the predominantly nocturnal woodlice *P. scaber* and *Oniscus asellus* are morphologically plastic, undergoing a striking diurnal cycle during which the contents of the apical cytoplasm, including the Fe inclusions and lipid, are extruded at the end of the dark phase and well into the light phase in a manner reminiscent of apocrine secretion; this is followed by gradual restitution beginning towards the end of the light phase and completed during the dark phase when the cytoplasm becomes fully recharged. Thus, according to Hames & Hopkin [36], there appears to be a continuous daily cycle of Fe acquisition and excretion from B cells. By contrast, Lešer *et al.* [29], in a less temporally resolved study, also observed B-cell lipid droplet extrusion in *P. scaber* that did not appear to follow a distinctive daily pattern. If daily B-cell rhythmicity as described by Hames & Hopkin [36] does occur, it begs fundamental questions regarding either the metabolic wastefulness or metabolic functions of wholesale release of lipid and mineralized Fe into the midgut lumen. On the other hand if, as according to Lešer *et al.* [29], it does not occur, we are still left with unanswered questions relating to the chemical states and possible metabolic interactivity of Cu and Fe within the contiguous S and B cells.

The broad aim of this study was the *in situ* characterization of the distinct Cu-phile and Fe-phile organelles in two specialized epithelial cell types in order to better understand how transition metal storage and metabolism in woodlice contribute to their success as land colonizers, and also to explore the possibility that these organelles might provide tractable model systems for probing vital aspects of transition metal speciation and interactions at the subcellular level. Of all the available bio-imaging techniques, high-brightness and highly coherent synchrotron-based X-ray fluorescence (XRF) microscopy is uniquely capable of providing the necessary analytical sensitivity, spatial resolution and ability to determine metal oxidation states [15,16,37] to pursue these goals. Our study had a core specific aim: to determine *in situ* the oxidation states and ligand-binding speciation of Cu and Fe in the S and B cells of the hepatopancreas in laboratory-acclimated woodlouse (*O. asellus*) at two extreme nodes of the presumptive daily B-cell extrusion/restitution cycle, using preparative procedures that preserve simultaneously the integrity of cellular morphology and chemistry. We undertook an adjunct experiment using light microscope-based morphometry to determine the presence of a daily wave of apocrine secretory activity in the B cells of our cultured woodlice.

## 2. Material and methods

### 2.1. Whole hepatopancreas elemental analyses

Pooled hepatopancreas tubules (each replicate dissected from five individual *Oniscus asellus*) were digested in 2 ml boiling 16N HNO_3_ on a sand bath. Digests were made up to 10 ml with ultrapure water, and analysed in a JY Horiba Ultima-2 inductively coupled plasma optical emission spectrometer (ICP-OES). Five replicates of hepatopancreas were collected at 02.00 and 12.00 h, these intervals corresponding with the times at which the tubules for synchrotron-based imaging and analysis were taken. Analyses of a standard tissue (marine mussel, GBW 08571; State Bureau of Technical Supervision, China) indicated that the combined tissue-processing and analytical protocols yielded values within 8% of expectations.

### 2.2. Hepatopancreas morphometry

For the morphometry experiment, adult inter-moult woodlice (*O. asellus*; Crustacea, Isopoda, Oniscidae) were collected from an unpolluted reference site at Coopers Field, Bute Park, Cardiff, UK (NGR 317819 176785, 51°29′20.4″ N, 3°11′20.4″ W). Eight individuals were placed into each of 12 plastic pots (8 cm diameter, 5 cm high) with perforated lids. Dry, heat-sterilized soil from the sampling site had already been inserted into the pots to a depth of 2 cm and then moistened thoroughly with deionized water. Leaf litter (*Acer pseudoplanatus*) from Coopers Field was added to the pots as a food source. Soil moisture and food were checked at weekly intervals during the acclimation period, and replenished as required. The woodlice were maintained in their pots for four weeks in a Binder GmbH incubator (Tuttlingen, Germany) at 20°C, 75% humidity, and a 12 L : 12 D regime (light on at 11.45, light off at 23.45). After the four-week acclimation period, pots containing woodlice were removed from the incubator at 2-hourly intervals for 24 h. Single hepatopancreas tubules freshly dissected from six individuals at each sampling interval were fixed for at least 24 h in 10% neutral-buffered-formalin. After washing in phosphate-buffered saline, groups of six tubules were embedded and aligned in parallel in warm 1% agarose within proprietary cryo-moulds. (Grouping tubules in this way significantly reduced sectioning and staining effort, and thus efficiently facilitated the replication necessary for morphometric analysis.) The solidified agarose blocks were dehydrated in ethanol, wax-embedded, sectioned at a thickness of 8 µm in the mid-regions of the aligned tubules, mounted on glass slides, and stained in haematoxylin and eosin (H&E) for imaging and morphometric analysis using a Leica DMR light microscope interfaced with a computer furnished with Leica LAS imaging software. The samples collected at 10.00 and 14.00 h were inadvertently lost during histological processing. Epithelial thickness in each tubule was determined using the modelling approach as described by Lešer *et al.* [29].

Some unfixed tubules from randomly chosen individual woodlice were embedded within proprietary cryo-moulds in Optimal Cutting Temperature compound (OCT; Agar Scientific UK), frozen in liquid N_2_, and sectioned at 10 µm thickness in a Bright OFT5000 cryostat (chamber temperature of −20°C) in OCT-filled cryo-moulds, for laser confocal imaging. These cryostat sections were stained for DNA with DAPI (*E*_x_ and *E*_m_ maxima = 358 nm and 461 nm, respectively; scanned using 495 nm laser line) and actin with Alexa Fluor^®^ 488 nm phalloidin (*E*_x_ and *E*_m_ maxima = 495 nm and 519 nm, respectively; scanned using 488 nm laser line). Cuprosomes were imaged in reflectance mode (488 nm, with a 10 nm detection band).

### 2.3. µ-Focus XAS *in situ* measurements in thin sections

In this experiment, woodlice were sampled from a standard laboratory culture in a controlled environment room (20°C, 75% humidity) under a 12 L : 12 D regime (light on at 06.00, light off at 18.00). Hepatopancreas samples for microfocus analyses were dissected and immediately processed from woodlice collected at 12.00 and 02.00 h (i.e. 6 h into the light and dark phases, respectively). These temporal ‘nodes' were initially chosen because they corresponded approximately to the mid-extrusion and mid-restitution phases of the diurnal apocrine cycle within the hepatopancreas as described by Hames & Hopkin [36]. The preparative procedure used was compared in a previous microfocus study on earthworms [38] with cryo-sectioning of unfixed fresh tissues, and was deemed to maintain faithfully both the morphological and compositional fidelity of tissues and cells to degrees permitting meaningful microfocus imaging, mapping and analysis. It involved gentle fixation for approximately 3 days in 70% alcohol of one or more tubules from individual woodlice, followed by glycol methacrylate embedding and transverse sectioning in the range of 2–10 µm. Sections were mounted on 25 × 50 mm spectroscopic-quality Spectrosil 2000^®^ or fused quartz Vitreosil 077^®^ glass slides (UQG Optics Ltd., Milton, Cambridge). Slides were inserted into the standard Diamond I18 beamline sample holder, orientated and imaged under brightfield illumination, then subjected to metal mapping and µ-focus spectroscopy, with different acquisition times and levels of spatial resolution, almost exclusively at ambient temperature.

XAS data were collected on beamline I18 at Diamond Light Source using a Si(111) double crystal monochromator, and the Kirkpatrick-Baez focusing mirrors, which provide a 3 µm spot size, were also used to remove harmonic contamination [39]. Calibration spectra for Cu, Fe and Zn foils were recorded in transmission mode. Data for the samples were collected in fluorescence mode using an Ortec (Oakridge, USA) 9-element Ge detector. Elemental distribution maps for Cu, Fe and Zn were acquired and processed essentially as described previously for Pb and Zn in sectioned earthworm tissues [40]; these were used to determine the areas to collect the XAS spectra. The XAS data were reduced in the program Athena [41], X-Ray Absorption Near Edge Structure (XANES) data were modelled as linear combinations of the XANES spectra of selected standards, collected previously and energy corrected using the calibration spectra [42]. The Extended X-ray Absorption Fine Structure (EXAFS) data were analysed using exact curved wave theory [43] in DL-Excurv [44]. Phaseshifts were derived in the program from *ab initio* calculations using Hedin–Lundqvist potentials and von Barth ground states [45]. The data were fitted for each sample by defining a theoretical model and comparing the calculated EXAFS spectrum with the experimental data. Shells of backscatterers were added around the absorber atom and by refining an energy correction E_f_ (the Fermi energy), the absorber–scatterer distance and the Debye–Waller factor for each shell, a least-squares residual (the *R*-factor [46]) was minimized. For fits with two shells, a reduced *χ*^2^-statistical test was used to check that inclusion of the additional fitting parameters was justified.

The stability under beam irradiation of the Cu, Fe and Zn signals was evaluated by collecting a series of consecutive XANES spectra under standard operational conditions from selected locations in S and B cells in the resin-impregnated tissue sections.

Some µXRF maps for P K-edge (2014 eV) and S K-edge (2308 eV) were acquired using a four element Si drift detector with a beryllium window (Hitachi Inc.) positioned close to the specimen. The relatively strong Ar K-edge (3.203 eV) signal from ambient air interferes with the P and S emissions; it was reduced but not eliminated by enclosing the specimen and detector inside a bespoke bag under flowing (300–400 ml min^−1^) He to give a largely He environment during analysis. No µXANES scans were collected at either the P or S K-edge. µXRF maps for these two anionic elements were used qualitatively to determine their co-distributions with the transition metals of interest.

## 3. Results

### 3.1. Elemental composition of whole hepatopancreas

There were no significant differences in the Cu, Fe, Zn and P concentrations in the hepatopancreas tubules of woodlice sampled at 02.00 and 12.00 h (table 1). Cu was the dominant transition metal with a concentration approximately ×6.5 higher than Fe, and approximately ×2 higher than Zn in these physiologically ‘normal’ samples. The concentrations of each of the Group 2 elements analysed (Mg, Ca, Sr, Ba) tended to be higher during the day (12.00 h) compared with the night (02.00 h); interestingly, metabolically essential Mg and Ca are recognized as predominantly extracellular in distribution. The concentration of K, a predominantly intracellular electrolyte, did not differ at the two time points.

**Table 1.** Elemental concentrations (µg/g dry weight) in pooled samples of hepatopancreas from *O. asellus* sampled from a reference site (Pontcanna) at two key time points. Data are presented as mean ± s.e. (*n* = 5 in each case).

treatment	Cu	Fe	Zn	P	Na	K	Mg	Ca	Sr	Ba
02.00 h	4948 ± 679	765 ± 86	2259 ± 516	1902 ± 152	12 108 ± 940	14 071 ± 951	2644 ± 176	4605 ± 959	114 ± 10	285 ± 20
12.00 h	4825 ± 405	711 ± 118	2679 ± 377	2091 ± 58	15 736 ± 1694	13 929 ± 810	2713 ± 323	5706 ± 939	140 ± 18	536 ± 138

### 3.2. Hepatopancreas morphometry

**Figure 1.**
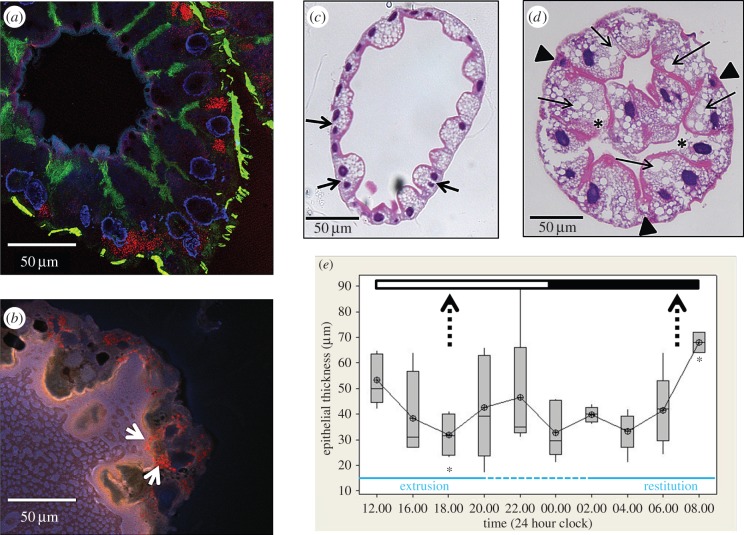
Morphology and morphometry of the hepatopancreas of *O. asellus*. (*a*) Confocal image of an unfixed, cryostat-sectioned hepatopancreas tubule immunofluorescently stained to illustrate general cell architecture. Note that nuclei are stained blue (DAPI) and actin filaments green (Alexa Fluor^®^ 488 nm phalloidin). Cuprosomes (red) in the ‘S' cells were imaged in reflectance mode. The tubule was dissected from a woodlouse near the peak of its restitution phase. (*b*) Confocal image of part of an unstained cryostat section taken from alongside the section depicted in 1(*a*) showing autofluorescence only; no fluorescent markers were introduced. Punctate red staining within the cytoplasm of a B cell (white arrows) represents autofluorescent lipid-containing droplets (note that the lumen is the lighter, left-hand side of the image). This fluorescence had an unusually long Stokes shift, being excited by the UV laser and emitting in the red part of the spectrum. (*c*) Light micrograph of a transverse H&E-stained section of a tubule near the nadir of the extrusion phase; note the variability in the shapes of the binucelate B cells (arrows), but with all containing small lipid inclusions. (*d*) Light micrograph of a transverse H&E-stained section of a tubule near the climax of the restitution phase where the apical cytoplasm of the B cells is engorged with large lipid inclusions (arrows); note the B cells (asterisks) with disrupted apical membranes that are apparently undergoing apocrine secretion, and the S cells (arrowheads). (*e*) Box-plot of the modelled epithelial thickness of the hepatopancreas of acclimated woodlice measured (*n* = 6) at regular intervals during a 24 h period; the dark and light phases are depicted at the top, and the observed extrusion and restitution phases depicted at the bottom (with the ‘intermediate’ region where hepatopancreas morphology was variable indicated by a broken line); the broken vertical arrows pinpoint the equivalent physiological periods identified by Hames & Hopkin [36] where restitution bottoms out (left) and extrusion climaxes (right); the asterisks indicate that the two points in our morphometric dataset nearest the Hames and Hopkin physiological transition regions are significantly different (Mann–Whitney, *p* < 0.05).

Epithelial thickness in the woodlouse hepatopancreas essentially reflects the height of the B cells protruding into the tubule lumen (figure 1*a,b*). Morphometric analysis at 2-hourly intervals provided evidence of a diurnal pattern of extrusion/restitution change in epithelial thickness (Kruskal–Wallis, *p* < 0.05) in woodlice acclimated to the experimental light regime (figure 1*c–e*). From the middle of the dark period (04.00 h), the mean epithelial thickness increased from its nadir of 33.2–68.0 µm at the end of the dark period (08.00 h). The B cells subsequently appeared to be more-or-less fully charged from around the night/day transition, rising to a peak at midday (12.00 h), from which point the thickness parameter fluctuated (reflecting considerable B-cell asynchrony, and inter-individual variability) but, nevertheless, tended to decline until the cytoplasmic restitution was initiated at night. Direct measurements of B-cell height confirmed this cyclical diurnal pattern (data not shown). A reversed light regime experiment (i.e. switching *O. asellus* from light : dark to dark : light over a four-week acclimation period) provided further evidence of rhythmicity; a distinct, albeit incomplete, shift in the pattern of cell morphology was observed with the extruded cells firmly confined to the light period and restitution peaking in the dark period (electronic supplementary material, figure S1).

### 3.3. µXAS *in situ* measurements: Cu, Fe and Zn K-edges in thin sections

**Figure 2.**
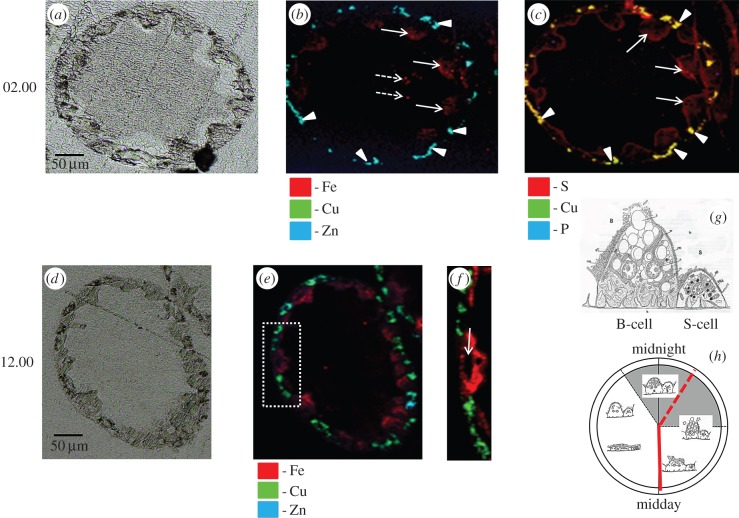
µXRF maps of element distributions in unstained methacrylate-embedded thin mid-tubule sections of woodlouse hepatopancreas. (*a*) Light micrograph of a transverse section from a woodlouse sampled at 02.00 h (i.e. the beginning of B-cell restitution during darkness). Note that the morphology of the sections used for µXRF is relatively unclear due to a lack of differential contrast in unstained, methacrylate-embedded sections. (*b*) Superimposed Cu, Fe and Zn µXRF maps of the section depicted in (*a*); note the co-distribution of Cu and Zn in S cells (arrowheads), and the distribution of diffuse Fe mainly in B-cell apical cytoplasm that might be undergoing blebbing (arrows), and some Fe signal within the tubule lumen (broken arrows). (*c*) Superimposed Cu, P and S µXRF maps of the section depicted in (*a*); note the co-distribution (yellow) of Cu and S in S cells (arrowheads), and the mainly apical distribution of P in B cells (arrows). (*d*) Light micrograph of a mid-tubule transverse section from a woodlouse at 12.00 h (i.e. at the beginning of B-cell extrusion during the day). (*e*) Superimposed Cu, Fe and Zn µXRF maps of the section depicted in (*d*); note that the transition metal distribution patterns are similar to those seen in (*b*). (*f*) Expanded view µXRF maps for the region delineated by a broken-lined rectangle in (*e*); note that Fe is distributed above and below the nuclear plane (arrow) of the prominent B cell, albeit mainly in the apical cytoplasm. (*g*) and (*h*) are schematic diagrams derived from Hames & Hopkin [36], illustrating the gross difference between B- and S-cell morphologies (*g*) and showing a ‘clock’ summarizing the diurnal cycle of apocrine secretion in B cells in relation to the light : dark regime with our two XAS sampling points superimposed upon it (*h*).

The µXRF maps more so than the corresponding unstained sections demonstrate that, in a given transverse optical plane, there is a fundamental symmetry in the arrangement of the two epithelial cells in the hepatopancreas, with each B cell flanked by two S cells (figure 2; electronic supplementary material, figures S2 and S3). µXRF maps indicate that Cu is concentrated as focal intracellular deposits in the basal cytoplasm of S cells in hepatopancreas tubules sampled at both 02.00 and 12.00 h (figure 2*b,e,f*; electronic supplementary material, figures S2*b*, S2*c* and S3). By contrast, Fe displayed a dispersed distribution pattern within B cells at both time intervals, but with an apparent bias towards the apical cytoplasm near the interval (02.00 h) when extrusion approaches completion and switches to restitution (figure 2*b,e,f*; electronic supplementary material, figures S2*b*, S2*c* and S3). Some images and µXRF maps (especially figure 2*b*; electronic supplementary material, figures S3*a*, S3*b*) provide evidence for the formation of large apical blebs in a number of B cells, with traces of diffuse material of apparently similar composition free in the lumen (figure 2*b*; electronic supplementary material, figure S3*b*). Cu was co-distributed with S, while Fe was co-distributed with P (cf. figure 2*b,c*). Zn was almost exclusively associated with Cu within the S cells of *O. asellus* hepatopancreas (figure 2*b,e,f*; electronic supplementary material, figures S2*b*, S2*c* and S3). Although the spatial resolution of the images obtained from the synchrotron is limited, some µXRF maps (e.g. electronic supplementary material, figure S2*c*) give the impression that the Cu-rich cytoplasm of S cells extends under the basal regions of the neighbouring B cells, indicating a close morphological intimacy between the two cell types.

**Figure 3.**
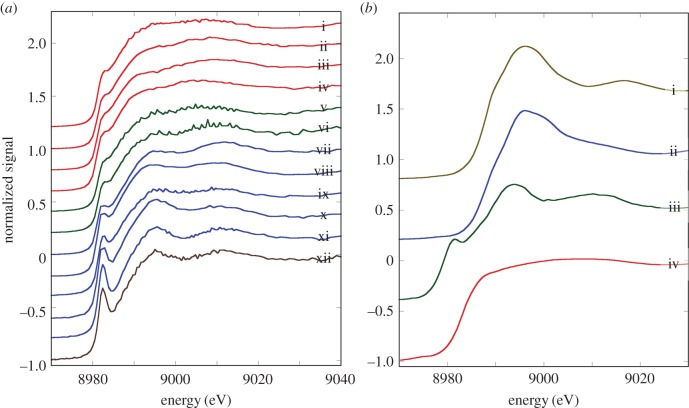
Comparative analysis of copper XANES spectra from S and B cells of the *O. asellus* hepatopancreas with model compounds. (*a*) Cu K-edge XANES spectra from: *O. asellus* S cells at 02.00 h (red; i–iv) and 12.00 h (green; v and vi); *O. asellus* B cells at 02.00 h (blue; vii–xi) and 12.00 h (brown; xii) clock time (see table 2 for the fit data). (*b*) Cu K-edge XANES spectra derived from model compounds: Cu(O_2_CCO_2_) (brown; i), Cu_3_(PO_4_)_2_ (blue; ii), Cu_2_O (green; iii) and CuS (red; iv).

**Figure 4.**
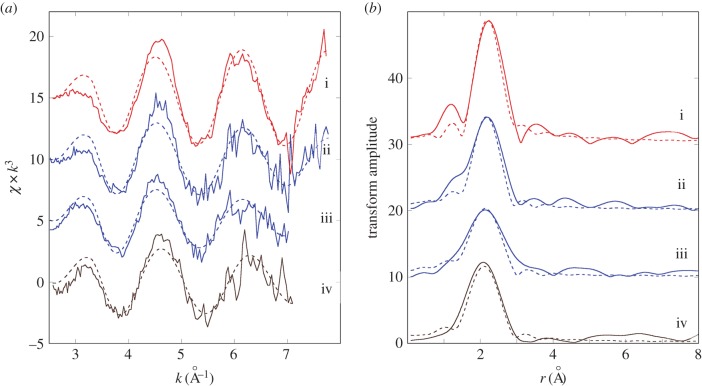
Determination of copper speciation from S and B cells of the *O. asellus* hepatopancreas. (*a*) Cu K-edge EXAFS data (solid lines) and fits (broken lines) from: an *O. asellus* S cell at 02.00 h (red; i); and B cells at 02.00 h (blue; ii and iii) and 12.00 h (brown; iv) (see table 3 for corresponding quantitative data). (*b*) Fourier transforms of the Cu EXAFS data (solid lines) and fits (broken lines) depicted in figure 4*a*.

The Cu K-edge XANES spectra exhibit three main features: a peak (or shoulder) on the absorption edge at 8982.5 eV, the edge crest at 8995 eV and a peak at 9011 eV (figure 3*a*). For the XANES spectra of the model compounds, only Cu_2_O shows the first peak (figure 3*b*), and its height in the spectra derived from methacrylate-embedded sections of woodlouse hepatopancreas correlates with the contribution from Cu_2_O in the XANES fitting (table 2). Moreover, the Cu edge values (8980 and 8981 eV) determined for spectra collected from S cells correspond well with literature values obtained for the maximum of the first derivative of XANES from a number of Cu(I) compounds (ranging from 8980.9 to 8983.1 eV) compared with Cu(II) compounds (8984.4 to 8988.0 eV) (electronic supplementary material, table S1). In the majority of cases, B-cell spectra displayed a higher contribution from the characteristic Cu(I) peak than did the spectra derived from S cells, perhaps suggesting that the B cells contain mixed Cu species albeit with a dominant S-bound phase. Calculated Cu K-edge values from B cells (8980.9 and 8981.0 eV) confirm the presence of reduced Cu(I) species in this cell type (electronic supplementary material, table S1). Both the XANES fitting (table 2) and the EXAFS fitting for four relatively noise-free spectra (figure 4*a,b* and table 3) showed that Cu, probably in its reduced Cu^+^ state, is mainly coordinated to S-donating ligands, with no indication of any outer shells. EXAFS indicated that there was no significant difference between the fit for an S cell at the time (02.00 h) roughly corresponding to the extrusion/restitution cross-over and the fits for B cells at the same time interval and near the restitution peak (12.00 h) (table 3). The data were not good enough to justify fitting the inner coordination sphere with a mixture of oxygen and sulfur scatterers.

**Table 2.** K-edge (Cu, Fe) XANES fitting of spectra derived from the two cell types in *O. asellus* hepatopancreas sampled at 02.00 (un-shaded rows) and 12.00 h (shaded rows).

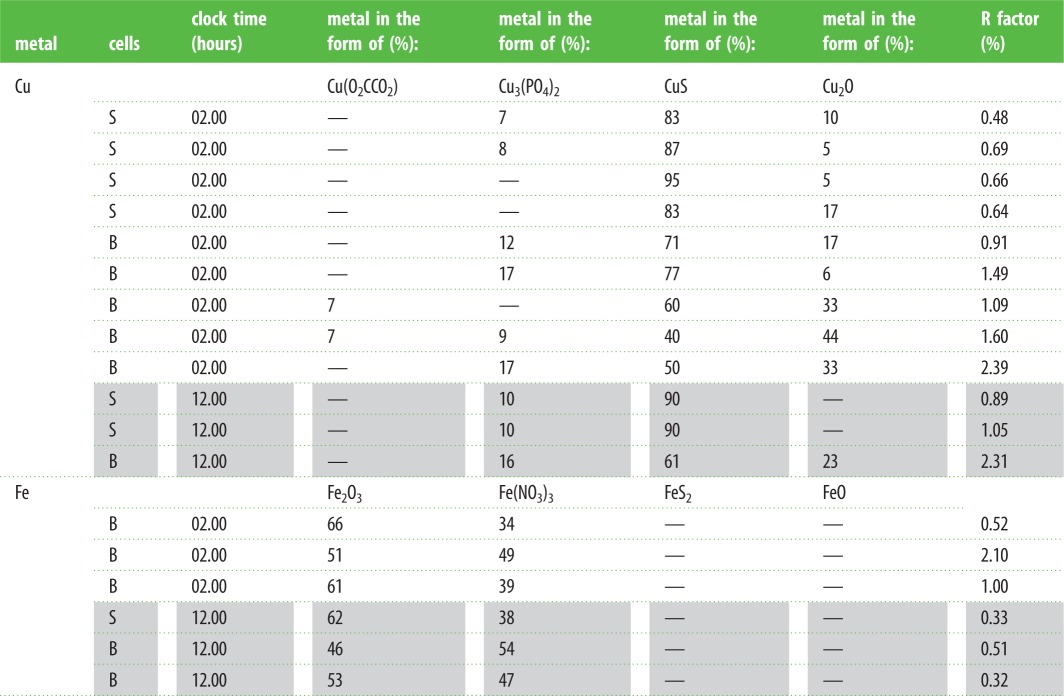

**Table 3.** K-edge EXAFS data (Cu, Fe) derived from the two cell types in *O. asellus* sampled at 02.00 (un-shaded rows) and 12.00 h (shaded rows).

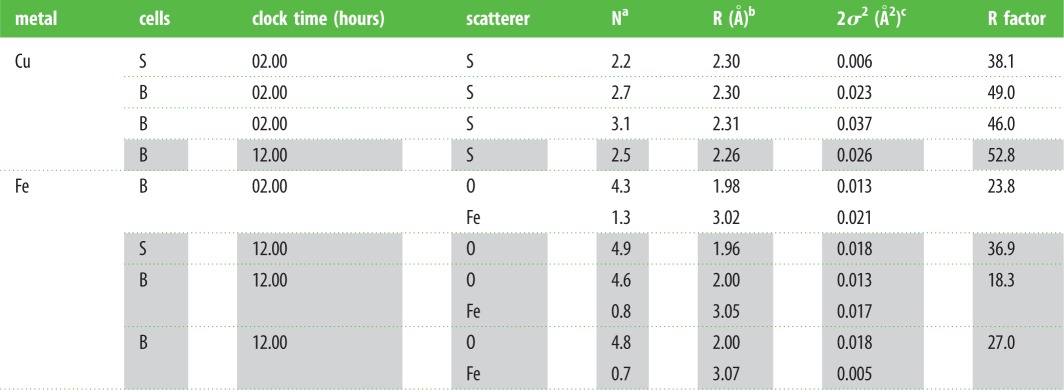

^a^Number of scatterers ±25%.

^b^Absorber–scatterer distance ±0.02 Å.

^c^Debye–Waller-type factor ±25%.

**Figure 5.**
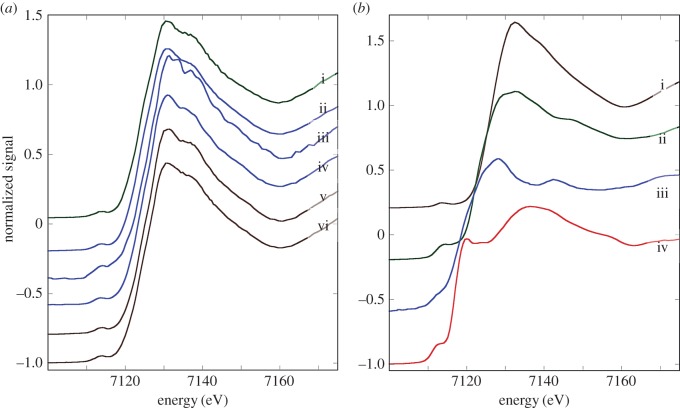
Comparative analysis of iron XANES spectra from S and B cells of the *O. asellus* hepatopancreas with model compounds. (*a*) Fe K-edge XANES spectra from: an *O. asellus* S cell at 12.00 h (green; i); and B cells at 02.00 h (blue; ii–iv) and 12.00 h (brown; v and vi) (see table 2 for the fit data). (*b*) Fe K-edge XANES spectra derived from model compounds: Fe(NO_3_)_3_ (brown; i), Fe_2_O_3_ (green; ii), FeO (blue; iii) and FeS_2_ (red; iv).

**Figure 6.**
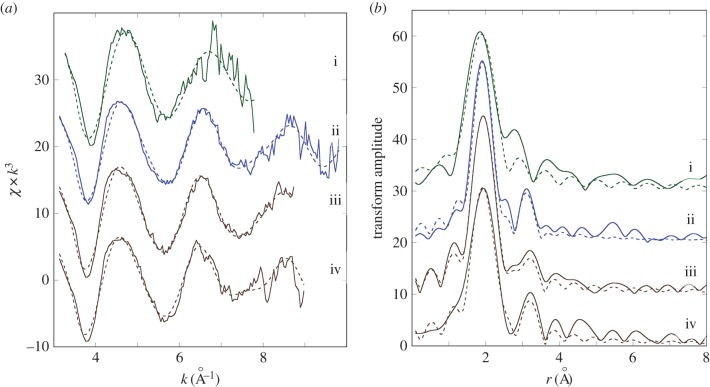
Determination of iron speciation from S and B cells of the *O. asellus* hepatopancreas. (*a*) Fe K-edge EXAFS data (solid lines) and fits (broken lines) from: an *O. asellus* S cell at 12.00 h (green; i), and B cells at 02.00 h (blue; ii) and 12.00 h (brown; iii and iv) (see table 3 for corresponding quantitative data). (*b*) Fourier transforms of the Fe EXAFS data (solid lines) and fits (broken lines) depicted in figure 6*a*.

The Fe K-edge XANES spectra were all very similar, with a pre-edge feature (1s-3d) at 7117 eV, the edge crest at 7131 eV and a peak at 7137 eV (figure 5*a*). The Fe XANES spectra from model compounds (figure 5*b*) most strongly corresponding to those from hepatopancreas thin sections were acquired from octahedrally oxygen-bound Fe^3+^ models, Fe_2_O_3_ and Fe(NO_3_)_3_ (cf. figure 5*a,b*). The XANES fitting (table 2) showed predominantly oxygen coordination for Fe, which was confirmed by EXAFS fitting (table 3 and figure 6*a,b*). The Fe–O bond length of approximately 2 Å is consistent with mainly six-coordinate Fe^3+^. An outer shell of Fe scatterers at approximately 3.05 Å could be fitted for the three EXAFS spectra derived from B cells (one at 02.00 h and two at 12.00 h), possibly implying the presence of an iron oxyhydroxide phase. The short usable data range of the single S-cell spectrum (12.00 h) prevented any outer shell fitting for this sample. There were no significant observable differences in Fe ligand-speciation binding between S and B cells, either in the XANES (table 2) or the EXAFS (table 3) datasets.

XANES and EXAFS spectra (data not shown) indicated that Zn is predominantly bound by O-donating ligands, with a relatively minor S-bound phase. This implies that while Zn is distinctly co-distributed with Cu within S cells (figure 2*b,e,f*; electronic supplementary material, figures S2*b*, S2*c* and S3), the cell may sequester redox-inactive Zn in two major pools. The electronic supplementary material, figure S2*c* provides spatial evidence that there is a shared Cu + Zn compartment, as well as separate Cu-only and Zn-only compartments in individual S cells. (The issue of Zn speciation under physiological and hyper-accumulation conditions is the subject of a separate detailed XAS study [47].)

**Figure 7.**
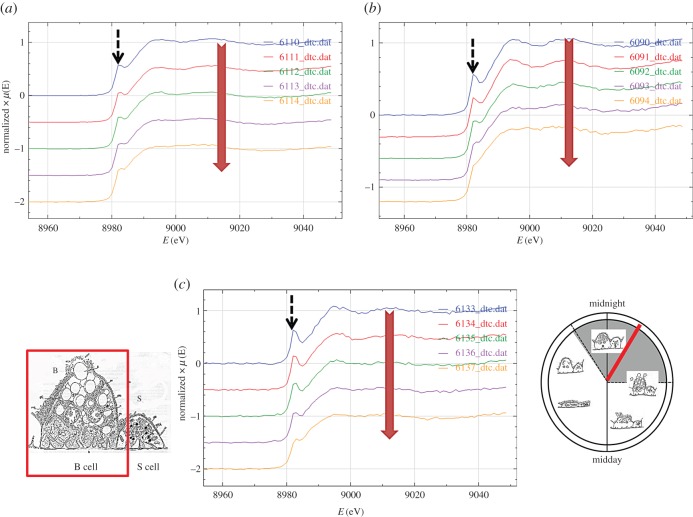
Radiation sensitivity of Cu speciation assessed by collecting a sequential series of five XANES spectra from given regions in three different B cells (*a–c*). The thin methacrylate-embedded sections were obtained from a woodlouse sampled at 02.00 h. In each panel, the large down-pointing vertical arrow indicates that the top spectrum was collected first and the lowest spectrum last. Note that the shoulder on the absorption edge at 8982.5 eV (broken arrows), while remaining visible, is progressively eroded from the third acquired spectrum onwards.

**Figure 8.**
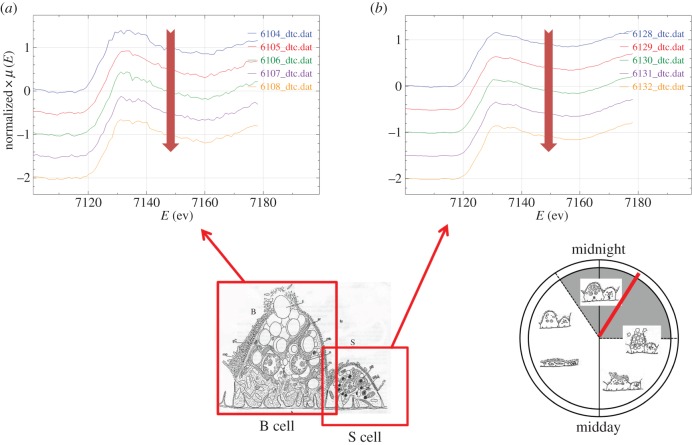
Radiation sensitivity of Fe speciation assessed by collecting a sequential series of five XANES spectra from given regions in a B cell (*a*) and in an S cell (*b*). The thin methacrylate-embedded sections were obtained from a woodlouse sampled at 02.00 h. In both panels, the large down-pointing vertical arrow indicates that the top spectrum was collected first and the lowest spectrum last. Note that successive spectra remain very similar.

Sequential analyses of a given spot on randomly selected hepatopancreas sections yielded some indication that the Cu XANES signal derived from B cells was initially stable under irradiation, but then showed some progressive erosion of the shoulder on the absorption edge at 8982.5 eV from the third spectrum onwards (figure 7*a–c*). Flattening after three or more acquisition scans of the Cu XANES signal derived from S cells was more subtle, possibly because the edge feature at 8982.5 eV was less pronounced (electronic supplementary material, figure S4*a–c*). Fe XANES spectra from both cell types were stable under repeated irradiation (figure 8*a,b*). Zn XANES spectra derived from S cells revealed some evidence of radiation-induced change in later scans (electronic supplementary material, figures S5*a* and S5*b*). Overall, these qualitative observations provide confidence in the biological veracity of the acquired qualitative and quantitative XAS data.

## 4. Discussion

Hames & Hopkin [36] qualitatively examined the fine structure of the S and B cells of two terrestrial isopod species (*O. asellus* and *P. scaber*) at hourly intervals over a 24 h period, and concluded that while the morphology of the S cells remained unchanged, B-cell morphology displayed the hallmarks of a diurnal pattern of apocrine secretion. By contrast, Lešer *et al*. [29] morphometrically examined the hepatopancreatic epithelium of *P. scaber* at four time points during a 24 h period and observed that neither epithelium thickness nor lipid droplet abundance differed significantly, although lipid droplets were concentrated conspicuously in B-cell apical cytoplasm 2 h into the light phase. Our findings on *O. asellus*, using the morphometric method described by Lešer *et al.* [29], were in general agreement with those of Hames & Hopkin [36]. Indeed, we also found that a cyclical apocrine pattern was evident, albeit less pronounced, in woodlice exposed to a reversed lighting regime for four weeks (electronic supplementary material, figure S1). It is difficult to reconcile the disparities relating to the presence or the absence of a B-cell apocrine cycle linked to a diurnal trophic pattern, but it is germane that according to Lešer *et al.* [29] the properties of the hepatopancreas epithelium are altered progressively after transference from favourable field conditions to laboratory culture where, for example, the diet is typically less heterogeneous. Other potential confounding factors are weak synchronicity in the B-cell population at certain time points, as well as a relatively high degree of inter-individual variation apparent in our morphometry dataset that probably reflects asynchronous feeding patterns. In any debate about the existence and pattern of apocrine secretion in isopod B cells, it is not inconsequential that these cells are binucleate. ‘Normal’ binucleate cells that are involved in forms of apocrine secretion are fairly common in insect taxa [48] where, according to Anhe & Azeredo-Oliveira [49], the additional genetic material is essential to drive cell restitution after the extensive secretory phase. Also pertinent is that a daily cycle of changes in haemolymph osmolality, ammonia and glucose levels linked to nocturnal feeding has been recorded in a semi-terrestrial isopod [50]. Moreover, Nakamura & Wright [51] recently described a diurnal cycle of glutamine storage and ammonia excretion in fully terrestrial isopods, probably associated with cyclical absorption and subsequent catabolism of dietary proteins.

In this study, whole hepatopancreas analyses, and *in situ* Cu and Fe K-edge µXAS analyses, did not reveal any striking temporal differences in metal content or ligand-binding speciation at the two selected time points (i.e. 6 h into the light and dark phases, respectively). Given the variations in cell morphology alluded to above, and the replication constraints imposed by the intrinsically low throughput of XAS micro-beam analysis, this may not be surprising.

Wieser [27] remarked that the woodlouse S cell is so densely packed with Cu-rich vesicles that it is difficult to envisage these small cells having the capacity to do anything other than sequester and store Cu. If we assume that the bulk of hepatopancreatic Cu resides in S cells, and that the estimated average volume fraction of these cells including the nucleus during the daily cycle is 20%, then extrapolating from tubule content of 5000 µgCu g^−1^ dry weight indicates that Cu represents approximately 2.5% (i.e. 25 000 µgCu g^−1^) of S-cell dry mass. To put this estimated value into a broader physiological context, liver and kidney with maximum values of approximately 60 µg g^−1^ dry weight are the most Cu-rich mammalian organs [52]. The stated theoretical Cu requirement of 44 µgCu ml^−1^ for haemocyanin in marine decapod crustaceans [53] implies that the hepatopancratic Cu reserves of terrestrial isopods, even if their physiological availability is only partial, probably exceed direct respiratory demands by orders of magnitude. But, in addition to acting as a respiratory pigment, haemocyanin in *P. scaber* can be activated to perform a role as a phenoloxidase [54]. Phenoloxidases facilitate the sclerotization of post-moult proteins and serve as key components of the primary immune cascade in arthropods [24]. They are also important in alimentary physiology; of direct relevance to this study, phenoloxidases have been shown to degrade phenolics and lignocelluloses in the isopod hindgut [21,55]. Because it has been postulated that only a fraction of phenoloxidase activity in woodlouse hindgut is attributable to gut flora [56], it is plausible that the hepatopancreas epithelia are another potential source of the Cu-containing enzyme. At the present time, the definitive molecular nature of the dominant Cu storage fractions in woodlouse hepatopancreas is not known. While not excluding the possibility that thiol-rich MTs may be involved in Cu storage, Donker *et al.* [34] favoured a role for haemocyanin and/or its metabolites. Since crystallographic study [57] shows that arthropod prophenoloxidase has the ‘canonical’ three di-nuclear Cu centre, with each Cu ion coordinated to non-thiol histidine, our µXANES and µEXAFS observations tend to suggest the involvement of Cu sequestering roles for MT-like peptides [58] in isopod S cells. It is pertinent that Engel & Brouwer [59] presented evidence that Cu-MT in a marine crustacean can transfer Cu to the active site of apohaemocyanin. Hopkin [60] stated unequivocally that wholesale voiding of cuprosomes from S cells into the lumen does not occur. The previously unsuspected detection of Cu in the apocrine B cells might lend credence to the hypothesis that the hepatopancreas exports Cu-containing molecules into the hepatopancreas lumen via this cell type, although our microfocus findings did not implicate a type 3 Cu protein.

Iron is the transition metal ion with the greatest variety of binding sites in proteins [58]. However, hardly any attention has previously been devoted to the coordination chemistry or functional aspects of Fe in woodlouse hepatopancreas. We detected Fe in both epithelial cell types, where its redox state and coordination chemistry was very different to that of Cu; Fe was always found as Fe^3+^ with predominantly O-coordination. To the extent that the hepatopancreas is a distinctly oxic environment [61], the Fe^3+^ oxidation state was not unexpected. The notion that Fe is regularly released along with lipid droplets from B cells into the alimentary lumen appears well-founded [36], whether a well-defined diurnal cycle of apocrine secretion occurs or not. Leaf litter rich in recalcitrant lignocelluloses is ingested by terrestrial woodlice and is ultimately digested by enzymes derived from litter-colonizing microflora and by endosymbionts [56] as well as from truly endogenous sources. Elegant work on wood-decaying fungi [62,63] shows that polysaccharide depolymerization by oxyradicals generated via Fenton-type reactions, involving Fe^2+^ and Fe^3+^ as well as Cu^2+^, is an important prelude facilitating access of enzymes to the dietary substrates. The micro-oxic/anoxic conditions prevailing in the radial centre of the posterior hindgut of woodlice [61] provide the reducing environment conducive to these reactions. However, the digestibility of cellulose is suppressed by Fe^3+^-mediated oxidation and cellulases can be inhibited by Fe^3+^ [64,65] but, again, reducing conditions in proximity to alimentary sites of lignocellulose digestion to monomeric products would promote enzyme activities. Polyphenols (tannins) ingested in large quantities by woodlice can not only potentially inhibit digestive enzyme activity by precipitation [66], but can also scavenge oxyradicals and block Fenton reactions by binding Fe^2+^ and Cu^1+^ [67,68]. It is known that the gut fluids of marine [69] and terrestrial isopods are rich in surfactants [70]. Surfactants are diverse in composition and sources, but they are often lipid derivatives [71]. Apart from facilitating the transport of hydrophobic digested lipids towards absorptive gut surfaces [72], they are known to counteract protein binding by polyphenols and, thus, can liberate the activities of phenoloxidases, catalases and other gut enzymes [70]. It is an intriguing possibility that lipid droplets regularly secreted by hepatopancreas B cells in harmony with the trophic cycle provide a continuous source of surfactant substrate and contribute, along with metallo-compound secretions, towards the efficient digestion of recalcitrant dietary macromolecules.

In conclusion, the findings of this study contribute to the general notion that alimentary function in woodlice such as *O. asellus* is a multifactorial network of interactions involving secretions from structurally and functionally differentiated regions of the invertebrate's alimentary canal, the activities of regionally specialized members of its microbiome, and ingested biotic and abiotic materials. More specifically, the findings call for a re-appraisal of the relationships between the hepatopancreatic B and S cells. These very specialized epithelial cells have to date been examined by a variety of microscopic and relatively insensitive microprobe analytical methods as if they are free-standing entities. High-resolution µXRF mapping with a high-brightness synchrotron beam suggests otherwise: a sounder hypothesis views them as morphologically integrated functional units. This view is supported by the intimacy of the highly infolded basal membranes of neighbouring cells [36] and the hitherto unsuspected ‘sharing’ of common Cu and Fe ligand-binding species within both cell types. The new paradigm envisages the partners forming these functional units having particular roles, with the S cells serving as dominant metal acquisition/storage hubs, while the B cells are primarily engaged in distribution logistics involving metals, metallo-compounds including pro-enzymes, and lipids. The paradigm should engender the formulation of novel hypotheses that can be tested with the full spectrum of contemporary bio-imaging and ‘omics' tools.

## Supplementary Material

Kille et al 2016 ESM

## Data accessibility

Raw spectral data associated with figures 2–8 and electronic supplementary material, figures S2–S5 are available from Dryad Digital Repository using the following link: http://dx.doi.org/10.5061/dryad.8734c.
