# Supplementary material for: ‘Venus trapped, Mars transits': Cu and Fe redox chemistry, cellular topography and in situ ligand binding in terrestrial isopod hepatopancreas
Source: Open Biol. 2016 Mar 2;6(3):150270. doi: 10.1098/rsob.150270 (PMC4821242; doi:10.1098/rsob.150270)
Supplement: Kille et al 2016 ESM [file rsob150270supp1.pdf]

Supplementary Material

Figure S1

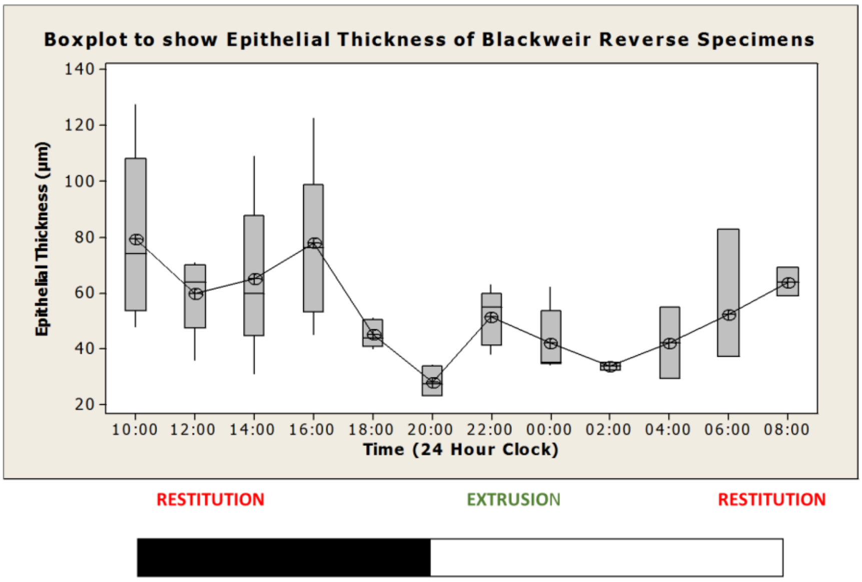

Figure S1. Box plot of the modelled epithelial thickness of the hepatopancreas of the woodlouse, *O. asellus*, acclimated for one month to a reversed Light:Dark regime. Note that the extrusion phase has shifted relative to clock time, so that it is predominantly associated with the Light Period, whilst restitution is completed in the Dark period. This pattern is evident despite the substantial inter-individual variability in epithelial thickness, especially during restitution. [‘n’ = 6 at each time interval.]

Figure S2

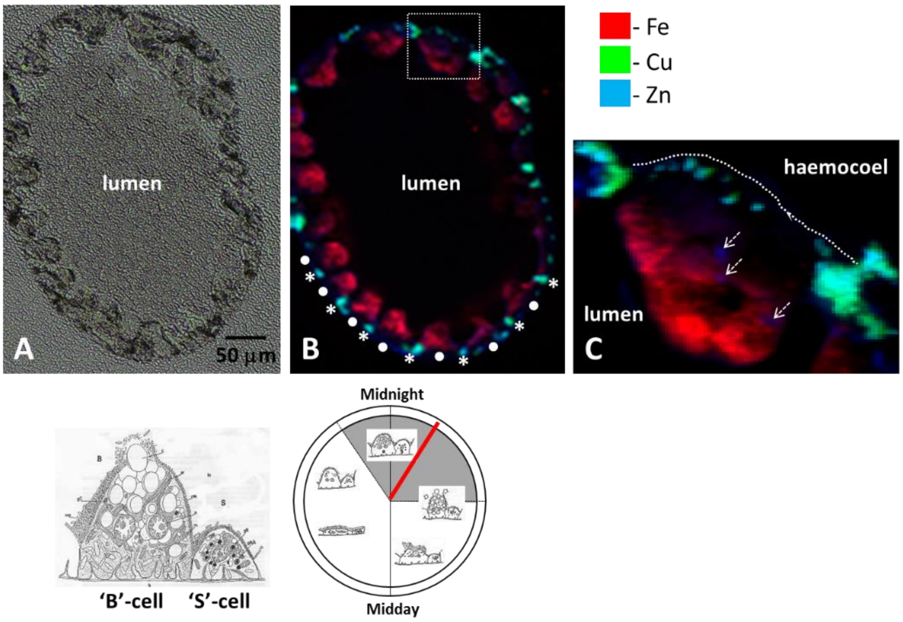

Figure S2. µXRF maps of element distributions in unstained methacrylate-embedded thin mid-tubule sections of woodlouse hepatopancreas sampled at 02:00 hours (i.e. the beginning of 'B'-cell restitution during darkness). **A.** Light micrograph of a transverse section. **B.** Superimposed Cu, Fe and Zn µXRF maps of the section depicted in S1A; note the regular 1:1 distribution of 'S'- (*) and 'B'-cells (•) in the tubule wall. **C.** Expanded view µXRF maps for the region delineated by a broken-lined rectangle inS2B; note the hallmarks of Cu/Zn-containing 'S'-cell cytoplasm 'under' the basal aspect of the 'B'-cell (dotted line), the co-distribution of Cu and Zn (light blue) as well as partitioning of Cu (green) and Zn (dark blue) in the 'S'-cell, and possibly some Zn-containing focal regions in the 'B'-cell (broken arrows).

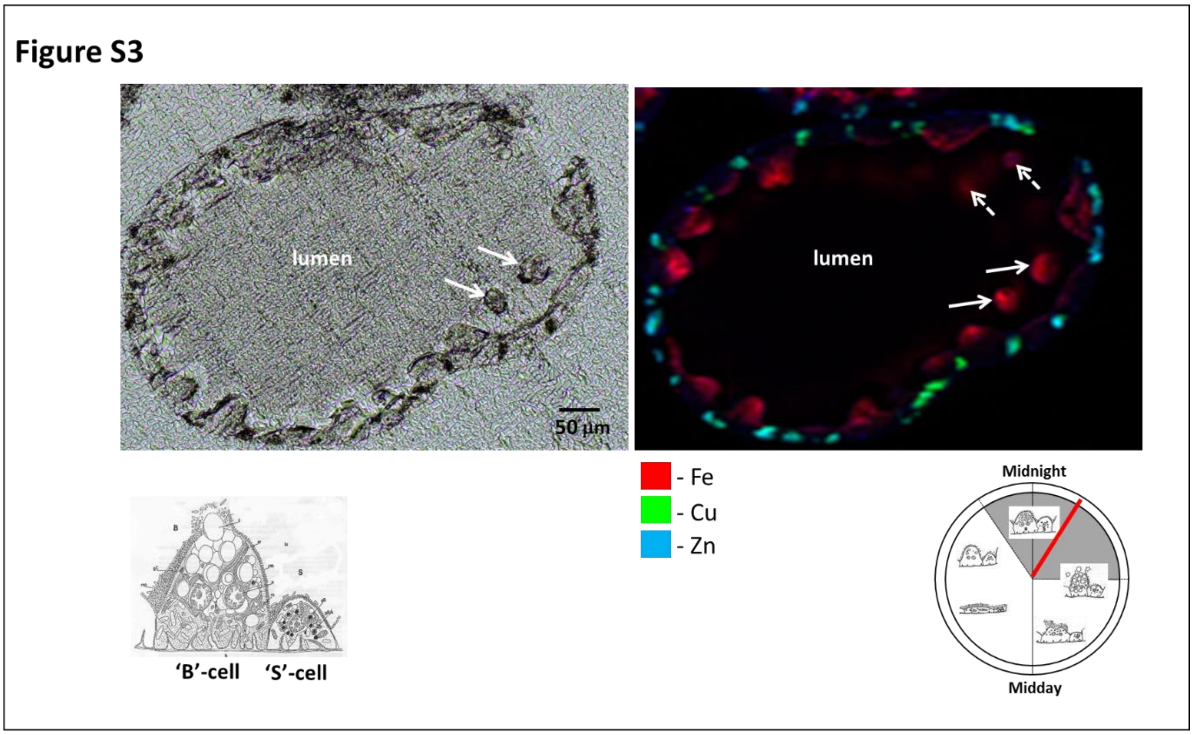

Figure S3. µXRF maps of element distributions in unstained methacrylate-embedded thin mid-tubule sections of woodlouse hepatopancreas sampled at 02:00 hours (i.e. the beginning of 'B'-cell restitution during darkness). **A.** Light micrograph of a transverse section; note two spherical structures (arrows) in the lumen that might represent the newly shed apical cytoplasm ('blebbing') of two adjacent 'B'-cells. **B.** Superimposed Cu, Fe and Zn µXRF maps of the section depicted in S3A; note the Fe-rich 'blebs' in the lumen (solid arrows) corresponding with the structures labelled in S3A, as well as two more diffuse entities (broken arrows) possibly representing 'older' blebs that were not discernible in the micrograph.

Figure S4

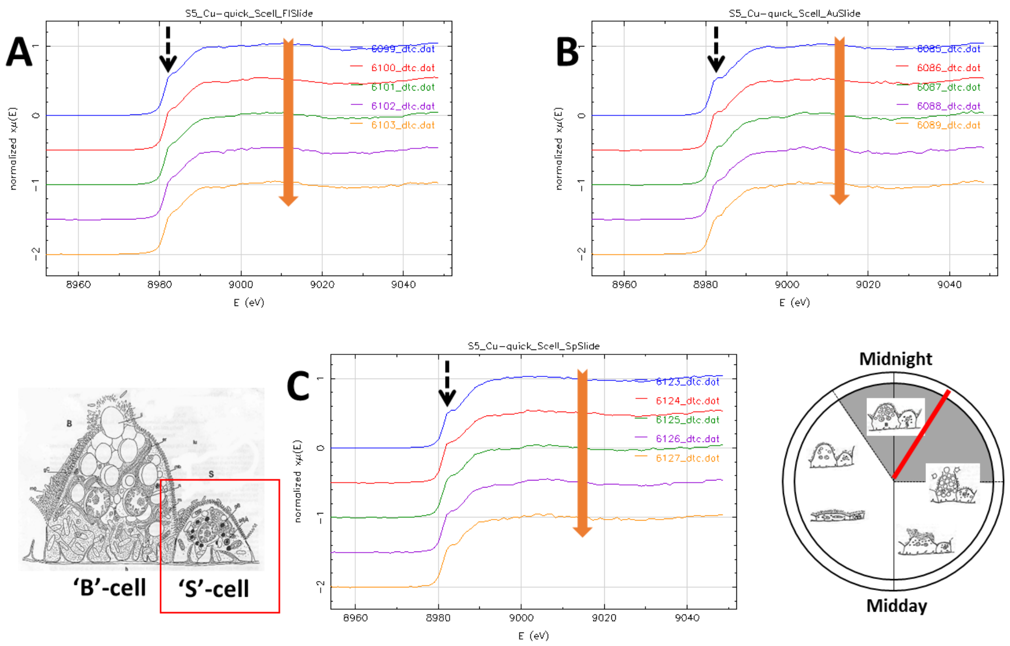

Figure S4. Radiation sensitivity of Cu assessed by collecting a sequential series of 5 XANES spectra from given regions in 3 different 'S'-cells (panels **A**, **B**, **C**). The thin methacrylate-embedded sections were obtained from a woodlouse sampled at 02:00 hours. In each panel, the large down-pointing vertical arrow indicates that the top spectrum was collected first and the lowest spectrum last. Note that the small shoulder on the absorption edge at 8982.5 eV (broken arrows) is slightly eroded from the third acquired spectrum onwards (cf. Fig. 7).

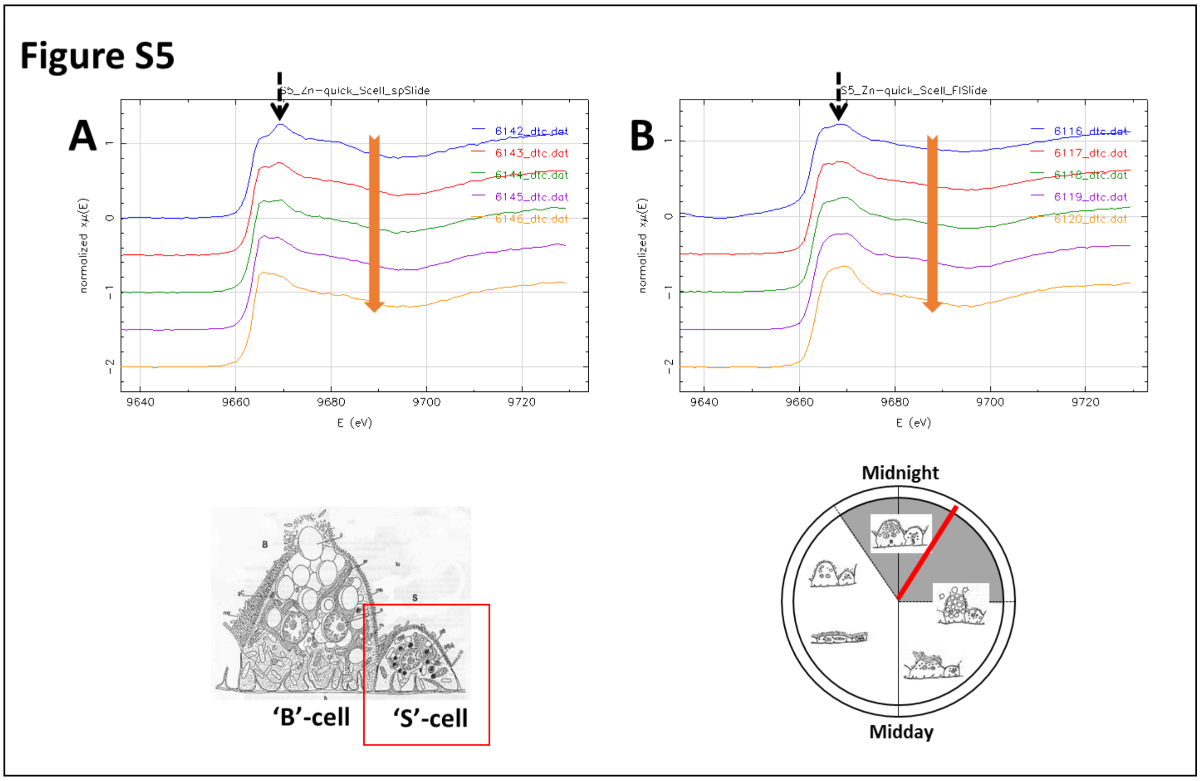

Figure S5. Radiation sensitivity of Zn assessed by collecting a sequential series of 5 XANES spectra from given regions in 2 different 'S'-cells (panels **A** and **B**). The thin methacrylate-embedded sections were obtained from a woodlouse sampled at 02:00 hours. In each panel, the large down-pointing vertical arrow indicates that the top spectrum was collected first and the lowest spectrum last. Note that the absorption edge peak at ~9670 eV (broken arrows) is slightly eroded from the 2nd acquisition onwards (**A**) and from the 4th onwards (**B**).

Table S1: Values of Cu K-edge positions relative to Cu metal at 8979.7 eV in pure Cu(I) and Cu(II) compounds, as well as calculated (Athena program) values in isopod 'S'- and 'B'-cells

Material	Edge Position (eV)
**Cu(I) species**	
Chalcocite, (Cu_2S)[1]	8980.9
Cuprous Chloride, (CuCl) [2]	8982
Covellite, (CuS)[1] *	8983.1
**Cu(II) species**	
[((Tetramethylethylenediamine)Cu)$_2$(S_2)$_2$](trifluoromethanesulfonate)$_2$[3]	8984.4
Azurite, $Cu_3(CO_3)_2(OH)_2$[1]	8986
Malachite, ($Cu_2CO_3(OH)_2$)[1]	8986
Chalcanthite, ($CuSO_4 \cdot 5H_2O$)[1]	8988
Tenorite, (CuO)[1]	8987
Cupric Chloride, ($CuCl_2$)[2]	8986
**'S'-cell** [Figure S4]	
Panel A	8981.0
Panel B	8980.0
Panel C	8981.0
**'B'cell** [Figure 7]	
Panel A	8980.9
Panel B	8981.0
Panel C	8981.0

*There is still debate over the oxidation state of covellite; theoretical calculations suggests it may be somewhere between Cu^{+} and $Cu^{1.166+}$. [4]

<u>Footnote</u>: There is an extensive literature on the XANES of Cu(I) and Cu(II) species, however because of the different transitions allowed by different Cu geometries and ligands, the variation of edge position with oxidation state is quite large.[5] Some of values reported for various Cu species edge position are shown in Table ST1 (above). It is also

known that XANES spectra of copper-based systems vary with co-ordination geometry due to the different possible orbital configurations ($4s^2$ $3d^9$ / $4s^1$ $3d^{10}$) that can be manifest.[5] The K-edge Fermi level is representative of the 1s – 4p transition; this is dependent on geometric and ligand effects as well as oxidation state. Thus while the Cu L3-edge edge positions is used to discuss oxidation state[6,7], in Cu K-edge XANES the presence, intensity and position of peaks in both the pre-edge and edge regions are more often used[8,9]. In particular Cu(I) compounds often have a shoulder around 8982.7 eV.[9]

References (Table S1 and Footnote – above): **[1]** Veiga, J. P. & M.O.Figueiredo, M. O. (2006). Copper blue in an ancient glass bead: a XANES study. *Appl Phys A* 83, 547–550. **[2]** Gaur, A. (2012). X-ray absorption fine structure (XAFS) studies of some copper compounds and complexes of biological importance. Ph. D. Thesis Vikram University, India. **[3]** Sarangi, R., York, J. T., Helton, M. E., Fujisawa, K., Karlin, K. D., Tolman, W. B., Hodgson, K. O., Hedman, B. & Solomon, E. I. (2008). X-ray absorption spectroscopic and theoretical studies on $(L)_2[Cu_2(S_2)_n]^{2+}$ complexes: disulfide versus disulfide(•1−) bonding. *J Am Chem Soc* 130, 676–686. **[4]** Conejeros, S., Moreira, I. D. P. R., Alemany, P. & Canadell, E. (2014). Nature of holes, oxidation states, and hypervalency in covellite (CuS). *Inorg Chem* 53, 12402−12406. **[5]** Kau, L. S., Spira-Solomon, D. J., Penner-Hahn, J. E., Hodgson, K. O. & Solomon E. I. (1987). X-ray absorption edge determination of the oxidation state and coordination number of copper: Application to the type 3 site in *Rhus vernicifera* laccase and its reaction with oxygen. *J Am Chem Soc* 109, 6433-6442. **[6]** Alp, E. E., Shenoy, G. K. , D. G. Hinks, D. G. , Capone, D. W. , Soderholm, L., Schuttler , H.-B., Guo, J. , Ellis, D. E., Montano, P.A. & Ramanathan, M. (1987). Determination of valence of Cu in superconducting La_2 (Sr, Ba) $Cu0_4$. *Phys Rev B* 35, 7199-7202. **[7]** Goha, S. W., Buckley, A.N., , Lamba, R. N., Rosenberg, R. A. & Moranc, D. (2006). The oxidation states of copper and iron in mineral sulfides, and the oxides formed on initial exposure of chalcopyrite and bornite to air. *Geochim Cosmochim Acta* 70, 2210-2228. **[8]** Shimizu, K., Maeshima, H., Yoshida, H., Atsushi Satsuma, A. & Hattori, T. (2001). Ligand field effect on the chemical shift in XANES spectra of Cu(II) compounds. *Phys Chem Chem Phys* 3, 862-866. **[9]** Clark, K., Penner-Hahn, J. E., Whittaker, M. M., & Whittaker, J. W. (1990). Oxidation-state assignments for galactose oxidase complexes from x-ray absorption spectroscopy. Evidence for copper (II) in the active enzyme. *J Am Chem Soc* 112, 6433-6434.
